# *cis*-Dichloridobis(1,10-phenanthroline-κ^2^ *N*,*N*′)manganese(II)–2,6-dihydroxy­benzoic acid–water (2/1/4)

**DOI:** 10.1107/S1600536808012427

**Published:** 2008-05-03

**Authors:** Qian Yang, Jing-Jing Nie, Duan-Jun Xu

**Affiliations:** a Department of Chemistry, Zhejiang University, People’s Republic of China

## Abstract

In the crystal structure of the title compound, [MnCl_2_(C_12_H_8_N_2_)_2_]·0.5C_7_H_6_O_4_·2H_2_O, the Mn^II^ complex assumes a distorted octa­hedral geometry formed by two chloride anions and two phenanthroline (phen) ligands. The 2,6-dihydroxy­benzoic acid mol­ecule is disordered about an inversion center. The face-to-face separations of 3.540 (11) and 3.429 (8) Å between parallel phen ligands indicate the existence of π–π stacking between adjacent Mn^II^ complexes. Uncoordinated water mol­ecules are linked with complex and dihydroxy­benzoic acid mol­ecules *via* O—H⋯Cl and O—H⋯O hydrogen bonds.

## Related literature

For general background, see: Su & Xu (2004 ▶). For related structures, see: McCann *et al.* (1998 ▶); Pan & Xu (2005 ▶).
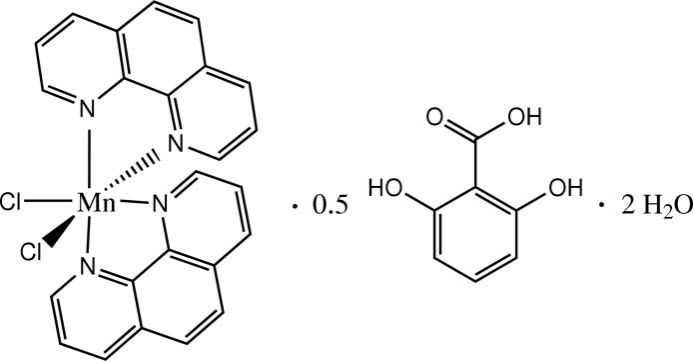


## Experimental

### 

#### Crystal data

[MnCl_2_(C_12_H_8_N_2_)_2_]·0.5C_7_H_6_O_4_·2H_2_O
*M* *_r_* = 599.34
Triclinic, 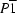

*a* = 9.757 (2) Å
*b* = 11.985 (3) Å
*c* = 13.261 (3) Å
α = 63.465 (17)°
β = 83.931 (18)°
γ = 76.819 (18)°
*V* = 1350.8 (6) Å^3^
*Z* = 2
Mo *K*α radiation
μ = 0.73 mm^−1^
*T* = 295 (2) K
0.42 × 0.36 × 0.20 mm

#### Data collection

Rigaku R-AXIS RAPID IP diffractometer
Absorption correction: multi-scan (*ABSCOR*; Higashi, 1995 ▶) *T*_min_ = 0.735, *T*_max_ = 0.860
14401 measured reflections
4680 independent reflections
3662 reflections with *I* > 2σ(*I*)
*R*_int_ = 0.029

#### Refinement

*R*[*F* ^2^ > 2σ(*F* ^2^)] = 0.059
*wR*(*F* ^2^) = 0.191
*S* = 1.08
4680 reflections
355 parameters
H-atom parameters constrained
Δρ_max_ = 0.55 e Å^−3^
Δρ_min_ = −1.16 e Å^−3^

### 

Data collection: *PROCESS-AUTO* (Rigaku, 1998 ▶); cell refinement: *PROCESS-AUTO*; data reduction: *CrystalStructure* (Rigaku/MSC, 2002 ▶); program(s) used to solve structure: *SIR92* (Altomare *et al.*, 1993 ▶); program(s) used to refine structure: *SHELXL97* (Sheldrick, 2008 ▶); molecular graphics: *ORTEP-3 for Windows* (Farrugia, 1997 ▶); software used to prepare material for publication: *WinGX* (Farrugia, 1999 ▶).

## Supplementary Material

Crystal structure: contains datablocks I, global. DOI: 10.1107/S1600536808012427/ng2452sup1.cif

Structure factors: contains datablocks I. DOI: 10.1107/S1600536808012427/ng2452Isup2.hkl

Additional supplementary materials:  crystallographic information; 3D view; checkCIF report

## Figures and Tables

**Table 1:** Selected bond lengths (Å)

Mn—N1	2.260 (3)
Mn—N2	2.328 (3)
Mn—N3	2.308 (3)
Mn—N4	2.275 (3)
Mn—Cl1	2.440 (2)
Mn—Cl2	2.4387 (13)

**Table 2:** Hydrogen-bond geometry (Å, °)

*D*—H⋯*A*	*D*—H	H⋯*A*	*D*⋯*A*	*D*—H⋯*A*
O1*W*—H1*A*⋯O3	0.84	2.06	2.834 (13)	152
O1*W*—H1*B*⋯O2*W*^i^	0.80	2.22	2.752 (6)	124
O2*W*—H2*A*⋯Cl1^ii^	0.85	2.09	2.904 (6)	162
O2*W*—H2*B*⋯Cl1	0.82	2.14	2.946 (4)	164
O1—H1*C*⋯O3	0.91	1.73	2.476 (18)	137
O2—H2*C*⋯O4	0.90	1.69	2.444 (19)	139
O4—H4*A*⋯O1*W*^iii^	0.88	2.28	2.886 (13)	125

Symmetry codes: (i) 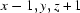; (ii) 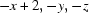; (iii) 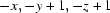.

**Fig. 1.**
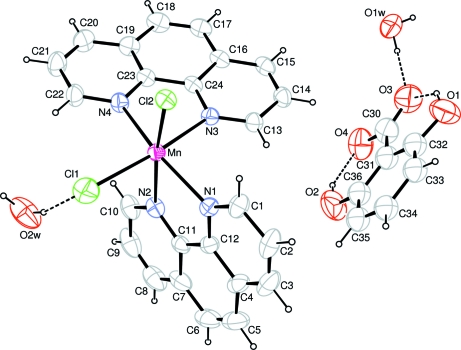
The molecular structure of the title compound with 30% probability displacement (arbitrary spheres for H atoms). Dashed lines indicate hydrogen bonding.

**Fig. 2.**
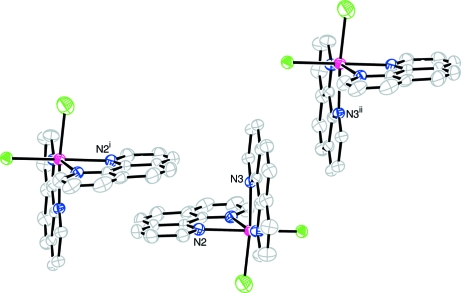
A diagram showing π-π stacking between phen ligands [symmetry codes: (i) 1 - x,1 - y,-z; (ii) 1 - x,-y,1 - z].

**Fig. 3.**
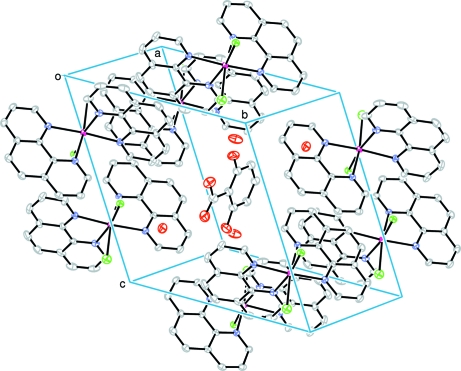
A packing diagram of the unit cell, H atoms and one of disordered components of dihydroxybenzoic acid have been omitted for clarity.

## supplementary crystallographic information

### Comment

As part of our ongoing investigation on the nature of π-π stacking (Su & Xu, 2004), the title compound incorporating 1,10-phenanthroline (phen) ligand has recently prepared and its crystal structure is reported here.

The crystal consists of Mn^II^ complex, uncoordinated dihydroxybenzoic acid and lattice water molecules (Fig. 1). The Mn^II^ complex assumes a distorted octahedral geometry formed by two Cl^-^ anions and two phen ligands (Table 1), similar to those found in crystal structure of *cis*-dichloro-bis(1,10-phenanthroline-κ^2^*N*,*N*')manganese(II) (Pan & Xu, 2005; McCann *et al.*, 1998). The two phen ligands of the complex are nearly perpendicular to each other with a dihedral angle of 83.50 (6)°. π-π stacking is observed in the crystal structure (Fig. 2). The face-to-face separation between parallel N2-containing phen and N2^i^-containing phen ligands is 3.540 (11) Å, while the face-to-face separation between parallel N3-phen and N3^ii^-phen ligands is 3.429 (8) Å [symmetry codes: (i) 1 - *x*,1 - *y*,-*z*; (ii) 1 - *x*,-*y*,1 - *z*].

The C30—O4 bond distance is significantly longer than C30—O3 bond distance (Table 1), which suggests that dihydroxybenzoic acid is a neutral molecule in the crystal. The uncoordinated dihydroxybenzoic acid molecule is located in a cavity formed by Mn^II^ complexes, and is close to an inversion center (Fig. 3). Therefore dihydroxybenzoic acid is disordered in the crystal with different spatial orientations. Lattice water molecules are linked with Mn^II^ complex and uncoordinated dihydroxybenzoic acid *via* O—H···Cl and O—H···O hydrogen bonding, respectively (Table 2 and Fig. 1).

### Experimental

Each reagent was commercially available and of analytical grade. MnCl_2_^.^4H_2_O (0.20 g, 1 mmol), 2,6-dihydroxybenzoic acid (0.15 g 1 mmol), 1,10-phenanthroline (0.39 g, 2 mmol) and Na_2_CO_3_ (0.053 g, 0.5 mmol) were dissolved in water-ethanol solution (15 ml, 10:5). The solution was refluxed for 4 h, and filtered after cooling to room temperature. Yellow single crystals were obtained from the filtrate after 2 d.

### Refinement

The dihydroxybenzoic acid is close to an inversion center and was refined with half site occupancy; the benzene ring was refined as a rigid group with the same displacement parameter for C atoms of the benzene ring. H atoms bonded to O atoms were located in a difference Fourier map and refined as riding in as-found relative positions with *U*_iso_(H) = 1.5*U*_eq_(O). Aromatic H atoms were placed in calculated positions with C—H = 0.93 Å, and refined in riding mode with *U*_iso_(H) = 1.2*U*_eq_(C).

### Crystal data

**Table:**

[MnCl_2_(C_12_H_8_N_2_)_2_]·0.5C_7_H_6_O_4_·2H_2_O	*Z* = 2
*M**_r_* = 599.34	*F*_000_ = 614
Triclinic, *P*1	*D*_x_ = 1.474 Mg m^−^^3^
Hall symbol: -P 1	Mo *K*α radiation λ = 0.71073 Å
*a* = 9.757 (2) Å	Cell parameters from 6825 reflections
*b* = 11.985 (3) Å	θ = 1.8–25.0º
*c* = 13.261 (3) Å	µ = 0.73 mm^−^^1^
α = 63.465 (17)º	*T* = 295 (2) K
β = 83.931 (18)º	Prism, yellow
γ = 76.819 (18)º	0.42 × 0.36 × 0.20 mm
*V* = 1350.8 (6) Å^3^	

### Data collection

**Table:**

Rigaku R-AXIS RAPID IP diffractometer	4680 independent reflections
Radiation source: fine-focus sealed tube	3662 reflections with *I* > 2σ(*I*)
Monochromator: graphite	*R*_int_ = 0.029
Detector resolution: 10.00 pixels mm^-1^	θ_max_ = 25.0º
*T* = 295(2) K	θ_min_ = 1.7º
ω scans	*h* = −10→11
Absorption correction: multi-scan(ABSCOR; Higashi, 1995)	*k* = −12→13
*T*_min_ = 0.735, *T*_max_ = 0.860	*l* = −15→15
14401 measured reflections	

### Refinement

**Table:**

Refinement on *F*^2^	Secondary atom site location: difference Fourier map
Least-squares matrix: full	Hydrogen site location: inferred from neighbouring sites
*R*[*F*^2^ > 2σ(*F*^2^)] = 0.059	H-atom parameters constrained
*wR*(*F*^2^) = 0.191	*w* = 1/[σ^2^(*F*_o_^2^) + (0.115*P*)^2^ + 0.6925*P*] where *P* = (*F*_o_^2^ + 2*F*_c_^2^)/3
*S* = 1.09	(Δ/σ)_max_ = 0.001
4680 reflections	Δρ_max_ = 0.55 e Å^−^^3^
355 parameters	Δρ_min_ = −1.16 e Å^−^^3^
Primary atom site location: structure-invariant direct methods	Extinction correction: none

### Special details

**Table:**

Geometry. All e.s.d.'s (except the e.s.d. in the dihedral angle between two l.s. planes) are estimated using the full covariance matrix. The cell e.s.d.'s are taken into account individually in the estimation of e.s.d.'s in distances, angles and torsion angles; correlations between e.s.d.'s in cell parameters are only used when they are defined by crystal symmetry. An approximate (isotropic) treatment of cell e.s.d.'s is used for estimating e.s.d.'s involving l.s. planes.
Refinement. Refinement of *F*^2^ against ALL reflections. The weighted *R*-factor *wR* and goodness of fit *S* are based on *F*^2^, conventional *R*-factors *R* are based on *F*, with *F* set to zero for negative *F*^2^. The threshold expression of *F*^2^ > σ(*F*^2^) is used only for calculating *R*-factors(gt) *etc*. and is not relevant to the choice of reflections for refinement. *R*-factors based on *F*^2^ are statistically about twice as large as those based on *F*, and *R*- factors based on ALL data will be even larger.

### Fractional atomic coordinates and isotropic or equivalent isotropic displacement parameters (Å^2^)

**Table:**

	*x*	*y*	*z*	*U*_iso_*/*U*_eq_	Occ. (<1)
Mn	0.81692 (6)	0.12805 (5)	0.21903 (4)	0.0452 (2)	
Cl1	0.9994 (2)	0.09801 (17)	0.08753 (14)	0.1086 (6)	
Cl2	0.93733 (10)	−0.05166 (9)	0.38297 (8)	0.0543 (3)	
N1	0.8875 (3)	0.2844 (3)	0.2384 (3)	0.0512 (8)	
N2	0.7264 (4)	0.3198 (3)	0.0676 (3)	0.0558 (8)	
N3	0.6117 (3)	0.1579 (3)	0.3159 (2)	0.0467 (7)	
N4	0.6685 (3)	0.0238 (3)	0.1901 (3)	0.0531 (8)	
O1	0.4543 (14)	0.3988 (12)	0.7173 (9)	0.132 (4)	0.50
H1C	0.3709	0.3781	0.7149	0.197*	0.50
O2	0.4410 (16)	0.5884 (14)	0.3174 (9)	0.146 (4)	0.50
H2C	0.3551	0.5690	0.3274	0.220*	0.50
O3	0.2390 (11)	0.4282 (12)	0.6173 (11)	0.135 (4)	0.50
O4	0.2332 (11)	0.5254 (11)	0.4305 (10)	0.127 (3)	0.50
H4A	0.1410	0.5328	0.4354	0.191*	0.50
O1W	0.0193 (5)	0.2911 (4)	0.6726 (4)	0.1147 (15)	
H1A	0.0606	0.3531	0.6449	0.172*	
H1B	−0.0422	0.3081	0.7116	0.172*	
O2W	0.8797 (5)	0.1644 (5)	−0.1317 (3)	0.1292 (19)	
H2A	0.9009	0.0907	−0.1291	0.194*	
H2B	0.9163	0.1605	−0.0767	0.194*	
C1	0.9627 (5)	0.2673 (5)	0.3244 (4)	0.0654 (12)	
H1	0.9819	0.1864	0.3842	0.078*	
C2	1.0141 (6)	0.3642 (6)	0.3295 (6)	0.0855 (16)	
H2	1.0653	0.3483	0.3915	0.103*	
C3	0.9875 (6)	0.4838 (6)	0.2409 (6)	0.0879 (17)	
H3	1.0224	0.5495	0.2416	0.105*	
C4	0.9086 (5)	0.5059 (4)	0.1508 (5)	0.0719 (13)	
C5	0.8757 (7)	0.6265 (5)	0.0563 (6)	0.099 (2)	
H5	0.9101	0.6943	0.0530	0.119*	
C6	0.7955 (8)	0.6450 (5)	−0.0291 (6)	0.101 (2)	
H6	0.7754	0.7256	−0.0891	0.121*	
C7	0.7401 (5)	0.5436 (5)	−0.0299 (4)	0.0750 (15)	
C8	0.6572 (7)	0.5591 (6)	−0.1146 (4)	0.096 (2)	
H8	0.6344	0.6384	−0.1758	0.115*	
C9	0.6079 (6)	0.4583 (7)	−0.1093 (4)	0.095 (2)	
H9	0.5503	0.4694	−0.1661	0.115*	
C10	0.6453 (5)	0.3365 (5)	−0.0163 (4)	0.0746 (14)	
H10	0.6135	0.2676	−0.0135	0.089*	
C11	0.7730 (4)	0.4222 (4)	0.0615 (3)	0.0560 (10)	
C12	0.8574 (4)	0.4022 (4)	0.1523 (3)	0.0529 (9)	
C13	0.5854 (4)	0.2205 (4)	0.3785 (3)	0.0559 (10)	
H13	0.6523	0.2638	0.3805	0.067*	
C14	0.4616 (5)	0.2253 (4)	0.4424 (4)	0.0623 (11)	
H14	0.4472	0.2706	0.4855	0.075*	
C15	0.3644 (5)	0.1629 (4)	0.4398 (3)	0.0615 (11)	
H15	0.2821	0.1644	0.4820	0.074*	
C16	0.3863 (4)	0.0947 (4)	0.3733 (3)	0.0530 (10)	
C17	0.2874 (4)	0.0270 (4)	0.3665 (4)	0.0643 (12)	
H17	0.2036	0.0266	0.4071	0.077*	
C18	0.3139 (5)	−0.0362 (5)	0.3023 (4)	0.0709 (13)	
H18	0.2469	−0.0780	0.2980	0.085*	
C19	0.4431 (4)	−0.0408 (4)	0.2403 (4)	0.0608 (11)	
C20	0.4783 (5)	−0.1079 (5)	0.1742 (4)	0.0751 (13)	
H20	0.4143	−0.1507	0.1669	0.090*	
C21	0.6039 (6)	−0.1109 (6)	0.1212 (5)	0.0800 (15)	
H21	0.6277	−0.1575	0.0794	0.096*	
C22	0.6973 (5)	−0.0441 (5)	0.1296 (4)	0.0664 (12)	
H22	0.7833	−0.0462	0.0921	0.080*	
C23	0.5434 (4)	0.0246 (4)	0.2452 (3)	0.0491 (9)	
C24	0.5137 (4)	0.0953 (3)	0.3123 (3)	0.0453 (8)	
C30	0.2957 (16)	0.4791 (16)	0.5273 (14)	0.098 (4)	0.50
C31	0.4417 (8)	0.4897 (11)	0.5167 (7)	0.0936 (16)	0.50
C32	0.5181 (10)	0.4401 (9)	0.6154 (6)	0.0936 (16)	0.50
C33	0.6607 (9)	0.4420 (9)	0.6100 (7)	0.0936 (16)	0.50
H33	0.7119	0.4088	0.6761	0.112*	0.50
C34	0.7267 (8)	0.4935 (10)	0.5060 (9)	0.0936 (16)	0.50
H34	0.8221	0.4948	0.5024	0.112*	0.50
C35	0.6503 (10)	0.5431 (9)	0.4073 (7)	0.0936 (16)	0.50
H35	0.6945	0.5775	0.3377	0.112*	0.50
C36	0.5077 (10)	0.5412 (9)	0.4127 (6)	0.0936 (16)	0.50

### Atomic displacement parameters (Å^2^)

**Table:**

	*U*^11^	*U*^22^	*U*^33^	*U*^12^	*U*^13^	*U*^23^
Mn	0.0442 (4)	0.0484 (4)	0.0457 (3)	−0.0130 (3)	0.0024 (2)	−0.0218 (3)
Cl1	0.1378 (15)	0.1000 (12)	0.0928 (10)	−0.0370 (10)	0.0080 (9)	−0.0421 (9)
Cl2	0.0508 (6)	0.0569 (6)	0.0510 (5)	−0.0092 (4)	−0.0020 (4)	−0.0203 (4)
N1	0.0456 (18)	0.0515 (19)	0.0585 (19)	−0.0100 (14)	0.0004 (14)	−0.0260 (16)
N2	0.0507 (19)	0.061 (2)	0.0484 (17)	−0.0017 (16)	−0.0026 (14)	−0.0216 (16)
N3	0.0403 (17)	0.0464 (17)	0.0496 (16)	−0.0062 (13)	0.0019 (13)	−0.0194 (14)
N4	0.0447 (18)	0.065 (2)	0.0572 (18)	−0.0139 (15)	0.0030 (14)	−0.0323 (17)
O1	0.140 (10)	0.152 (9)	0.093 (7)	−0.070 (8)	0.013 (6)	−0.030 (6)
O2	0.171 (12)	0.210 (13)	0.086 (7)	−0.077 (10)	0.002 (7)	−0.071 (8)
O3	0.103 (7)	0.172 (10)	0.165 (10)	−0.063 (7)	0.031 (7)	−0.095 (9)
O4	0.095 (7)	0.158 (9)	0.146 (9)	−0.001 (6)	−0.006 (7)	−0.091 (8)
O1W	0.141 (4)	0.098 (3)	0.126 (4)	−0.037 (3)	0.024 (3)	−0.068 (3)
O2W	0.099 (3)	0.146 (4)	0.077 (2)	0.022 (3)	0.002 (2)	−0.014 (3)
C1	0.062 (3)	0.068 (3)	0.078 (3)	−0.010 (2)	−0.013 (2)	−0.041 (2)
C2	0.070 (3)	0.097 (4)	0.122 (5)	−0.007 (3)	−0.015 (3)	−0.078 (4)
C3	0.067 (3)	0.089 (4)	0.144 (5)	−0.026 (3)	0.008 (3)	−0.079 (4)
C4	0.064 (3)	0.049 (3)	0.105 (4)	−0.015 (2)	0.026 (3)	−0.040 (3)
C5	0.099 (5)	0.055 (3)	0.127 (5)	−0.027 (3)	0.038 (4)	−0.028 (3)
C6	0.112 (5)	0.047 (3)	0.101 (4)	−0.009 (3)	0.040 (4)	−0.008 (3)
C7	0.070 (3)	0.059 (3)	0.061 (3)	0.005 (2)	0.018 (2)	−0.007 (2)
C8	0.098 (4)	0.085 (4)	0.057 (3)	0.019 (3)	0.004 (3)	−0.009 (3)
C9	0.083 (4)	0.128 (5)	0.050 (3)	0.025 (4)	−0.018 (2)	−0.035 (3)
C10	0.071 (3)	0.093 (4)	0.052 (2)	0.005 (3)	−0.011 (2)	−0.032 (2)
C11	0.053 (2)	0.049 (2)	0.052 (2)	−0.0009 (18)	0.0151 (18)	−0.0174 (18)
C12	0.045 (2)	0.047 (2)	0.064 (2)	−0.0090 (17)	0.0112 (18)	−0.0249 (19)
C13	0.055 (2)	0.056 (2)	0.058 (2)	−0.0078 (19)	0.0042 (18)	−0.028 (2)
C14	0.066 (3)	0.059 (3)	0.058 (2)	−0.001 (2)	0.007 (2)	−0.028 (2)
C15	0.049 (2)	0.060 (3)	0.056 (2)	0.003 (2)	0.0095 (18)	−0.017 (2)
C16	0.039 (2)	0.056 (2)	0.047 (2)	−0.0011 (17)	−0.0008 (16)	−0.0113 (18)
C17	0.042 (2)	0.073 (3)	0.067 (3)	−0.013 (2)	0.0020 (19)	−0.021 (2)
C18	0.047 (2)	0.089 (3)	0.074 (3)	−0.025 (2)	−0.005 (2)	−0.027 (3)
C19	0.051 (2)	0.070 (3)	0.063 (2)	−0.019 (2)	−0.0091 (19)	−0.026 (2)
C20	0.066 (3)	0.093 (4)	0.088 (3)	−0.033 (3)	−0.004 (2)	−0.050 (3)
C21	0.079 (3)	0.103 (4)	0.091 (3)	−0.030 (3)	0.003 (3)	−0.066 (3)
C22	0.065 (3)	0.086 (3)	0.071 (3)	−0.025 (2)	0.009 (2)	−0.052 (3)
C23	0.043 (2)	0.055 (2)	0.0457 (19)	−0.0085 (16)	−0.0057 (15)	−0.0180 (17)
C24	0.0361 (19)	0.049 (2)	0.0396 (17)	−0.0025 (15)	−0.0032 (14)	−0.0116 (16)
C30	0.076 (8)	0.114 (11)	0.116 (12)	−0.018 (8)	0.007 (8)	−0.062 (10)
C31	0.097 (4)	0.090 (3)	0.101 (4)	−0.021 (3)	0.013 (3)	−0.050 (4)
C32	0.097 (4)	0.090 (3)	0.101 (4)	−0.021 (3)	0.013 (3)	−0.050 (4)
C33	0.097 (4)	0.090 (3)	0.101 (4)	−0.021 (3)	0.013 (3)	−0.050 (4)
C34	0.097 (4)	0.090 (3)	0.101 (4)	−0.021 (3)	0.013 (3)	−0.050 (4)
C35	0.097 (4)	0.090 (3)	0.101 (4)	−0.021 (3)	0.013 (3)	−0.050 (4)
C36	0.097 (4)	0.090 (3)	0.101 (4)	−0.021 (3)	0.013 (3)	−0.050 (4)

### Geometric parameters (Å, °)

**Table:**

Mn—N1	2.260 (3)	C7—C11	1.408 (6)
Mn—N2	2.328 (3)	C8—C9	1.370 (9)
Mn—N3	2.308 (3)	C8—H8	0.9300
Mn—N4	2.275 (3)	C9—C10	1.425 (8)
Mn—Cl1	2.440 (2)	C9—H9	0.9300
Mn—Cl2	2.4387 (13)	C10—H10	0.9300
N1—C1	1.334 (5)	C11—C12	1.430 (6)
N1—C12	1.351 (5)	C13—C14	1.405 (6)
N2—C10	1.350 (6)	C13—H13	0.9300
N2—C11	1.370 (6)	C14—C15	1.348 (7)
N3—C13	1.319 (5)	C14—H14	0.9300
N3—C24	1.360 (5)	C15—C16	1.419 (6)
N4—C22	1.347 (5)	C15—H15	0.9300
N4—C23	1.355 (5)	C16—C24	1.410 (5)
O1—C32	1.351 (12)	C16—C17	1.428 (6)
O1—H1C	0.9109	C17—C18	1.344 (7)
O2—C36	1.307 (13)	C17—H17	0.9300
O2—H2C	0.9016	C18—C19	1.434 (7)
O3—C30	1.211 (17)	C18—H18	0.9300
O4—C30	1.305 (17)	C19—C20	1.405 (7)
O4—H4A	0.8817	C19—C23	1.409 (6)
O1W—H1A	0.8434	C20—C21	1.348 (7)
O1W—H1B	0.8012	C20—H20	0.9300
O2W—H2A	0.8461	C21—C22	1.385 (7)
O2W—H2B	0.8246	C21—H21	0.9300
C1—C2	1.396 (7)	C22—H22	0.9300
C1—H1	0.9300	C23—C24	1.449 (5)
C2—C3	1.376 (8)	C30—C31	1.445 (15)
C2—H2	0.9300	C31—C32	1.3900
C3—C4	1.381 (8)	C31—C36	1.3900
C3—H3	0.9300	C32—C33	1.3900
C4—C5	1.421 (8)	C33—C34	1.3900
C4—C12	1.432 (6)	C33—H33	0.9300
C5—C6	1.351 (10)	C34—C35	1.3900
C5—H5	0.9300	C34—H34	0.9300
C6—C7	1.443 (9)	C35—C36	1.3900
C6—H6	0.9300	C35—H35	0.9300
C7—C8	1.373 (8)		
			
N1—Mn—N4	158.51 (12)	N2—C11—C12	117.6 (3)
N1—Mn—N3	90.22 (12)	C7—C11—C12	120.3 (5)
N4—Mn—N3	73.23 (12)	N1—C12—C11	118.7 (4)
N1—Mn—N2	72.54 (12)	N1—C12—C4	121.2 (4)
N4—Mn—N2	92.82 (13)	C11—C12—C4	120.1 (4)
N3—Mn—N2	87.93 (11)	N3—C13—C14	123.4 (4)
N1—Mn—Cl2	97.72 (9)	N3—C13—H13	118.3
N4—Mn—Cl2	97.17 (9)	C14—C13—H13	118.3
N3—Mn—Cl2	94.48 (8)	C15—C14—C13	118.5 (4)
N2—Mn—Cl2	170.00 (10)	C15—C14—H14	120.7
N1—Mn—Cl1	98.05 (9)	C13—C14—H14	120.7
N4—Mn—Cl1	96.38 (9)	C14—C15—C16	120.6 (4)
N3—Mn—Cl1	167.55 (9)	C14—C15—H15	119.7
N2—Mn—Cl1	85.73 (9)	C16—C15—H15	119.7
Cl2—Mn—Cl1	93.61 (6)	C24—C16—C15	116.8 (4)
C1—N1—C12	118.4 (4)	C24—C16—C17	119.8 (4)
C1—N1—Mn	125.0 (3)	C15—C16—C17	123.4 (4)
C12—N1—Mn	116.5 (3)	C18—C17—C16	120.9 (4)
C10—N2—C11	118.8 (4)	C18—C17—H17	119.6
C10—N2—Mn	126.9 (3)	C16—C17—H17	119.6
C11—N2—Mn	114.0 (3)	C17—C18—C19	121.6 (4)
C13—N3—C24	118.4 (3)	C17—C18—H18	119.2
C13—N3—Mn	127.1 (3)	C19—C18—H18	119.2
C24—N3—Mn	114.3 (2)	C20—C19—C23	116.7 (4)
C22—N4—C23	117.9 (4)	C20—C19—C18	124.2 (4)
C22—N4—Mn	126.1 (3)	C23—C19—C18	119.1 (4)
C23—N4—Mn	115.9 (3)	C21—C20—C19	120.6 (4)
C32—O1—H1C	111.8	C21—C20—H20	119.7
C36—O2—H2C	111.9	C19—C20—H20	119.7
C30—O4—H4A	114.1	C20—C21—C22	119.5 (5)
H1A—O1W—H1B	105.4	C20—C21—H21	120.2
H2A—O2W—H2B	107.1	C22—C21—H21	120.2
N1—C1—C2	123.5 (5)	N4—C22—C21	122.5 (4)
N1—C1—H1	118.2	N4—C22—H22	118.7
C2—C1—H1	118.2	C21—C22—H22	118.7
C3—C2—C1	118.6 (5)	N4—C23—C19	122.7 (4)
C3—C2—H2	120.7	N4—C23—C24	117.9 (3)
C1—C2—H2	120.7	C19—C23—C24	119.4 (4)
C2—C3—C4	119.7 (5)	N3—C24—C16	122.3 (4)
C2—C3—H3	120.2	N3—C24—C23	118.6 (3)
C4—C3—H3	120.2	C16—C24—C23	119.2 (4)
C3—C4—C5	123.3 (5)	O3—C30—O4	123.6 (16)
C3—C4—C12	118.6 (5)	O3—C30—C31	123.0 (14)
C5—C4—C12	118.1 (6)	O4—C30—C31	113.3 (14)
C6—C5—C4	121.5 (6)	C32—C31—C36	120.0
C6—C5—H5	119.3	C32—C31—C30	117.6 (8)
C4—C5—H5	119.3	C36—C31—C30	122.4 (8)
C5—C6—C7	122.1 (5)	O1—C32—C31	121.1 (8)
C5—C6—H6	119.0	O1—C32—C33	118.7 (8)
C7—C6—H6	119.0	C31—C32—C33	120.0
C8—C7—C11	118.4 (6)	C32—C33—C34	120.0
C8—C7—C6	123.7 (5)	C32—C33—H33	120.0
C11—C7—C6	117.9 (5)	C34—C33—H33	120.0
C9—C8—C7	120.4 (5)	C35—C34—C33	120.0
C9—C8—H8	119.8	C35—C34—H34	120.0
C7—C8—H8	119.8	C33—C34—H34	120.0
C8—C9—C10	119.5 (5)	C36—C35—C34	120.0
C8—C9—H9	120.2	C36—C35—H35	120.0
C10—C9—H9	120.2	C34—C35—H35	120.0
N2—C10—C9	120.8 (6)	O2—C36—C35	117.5 (9)
N2—C10—H10	119.6	O2—C36—C31	122.5 (9)
C9—C10—H10	119.6	C35—C36—C31	120.0
N2—C11—C7	122.1 (5)		

### Hydrogen-bond geometry (Å, °)

**Table:**

*D*—H···*A*	*D*—H	H···*A*	*D*···*A*	*D*—H···*A*
O1W—H1A···O3	0.84	2.06	2.834 (13)	152
O1W—H1B···O2W^i^	0.80	2.22	2.752 (6)	124
O2W—H2A···Cl1^ii^	0.85	2.09	2.904 (6)	162
O2W—H2B···Cl1	0.82	2.14	2.946 (4)	164
O1—H1C···O3	0.91	1.73	2.476 (18)	137
O2—H2C···O4	0.90	1.69	2.444 (19)	139
O4—H4A···O1W^iii^	0.88	2.28	2.886 (13)	125
C3—H3···O1W^iv^	0.93	2.57	3.356 (9)	143
C5—H5···Cl1^v^	0.93	2.64	3.427 (7)	143
C9—H9···O1^vi^	0.93	2.41	3.275 (15)	154
C9—H9···O2^vii^	0.93	2.39	3.152 (15)	140
C15—H15···Cl2^viii^	0.93	2.82	3.674 (5)	153

Symmetry codes: (i) *x*−1, *y*, *z*+1; (ii) −*x*+2, −*y*, −*z*; (iii) −*x*, −*y*+1, −*z*+1; (iv) −*x*+1, −*y*+1, −*z*+1; (v) −*x*+2, −*y*+1, −*z*; (vi) *x*, *y*, *z*−1; (vii) −*x*+1, −*y*+1, −*z*; (viii) −*x*+1, −*y*, −*z*+1.
